# Selection of HMG-coenzyme A reductase inhibitors using multiattribute scoring tool

**DOI:** 10.1186/1471-2458-12-S2-A21

**Published:** 2012-11-27

**Authors:** Azuana Ramli, Syed Mohamed Aljunid, Saperi Sulong, Faridah Aryani Mohd Yusof

**Affiliations:** 1 United Nations University-International Institute for Global Health, Universiti Kebangsaan Malaysia Medical Centre, Jalan Yaacob Latiff, 56000 Kuala Lumpur, Malaysia; 2 Department of Health Information, Universiti Kebangsaan Malaysia Medical Centre, Jalan Yaacob Latiff, 56000 Kuala Lumpur, Malaysia; 3 Pharmaceutical Services Division, Ministry of Health, 46350 Petaling Jaya, Malaysia

## Background

Hypercholesterolaemia is a major risk factor for cardiovascular diseases. Treatment with HMG coenzyme A reductase inhibitors (statins) had been proven to reduce the risk. Due to high prevalence of hypercholesterolaemia, statins consumption is high. Statins available in Malaysia include atorvastatin, lovastatin, pravastatin, rosuvastatin, simvastatin and fluvastatin. All except one (fluvastatin) have piled up the MOH Drug Formulary (DF); hence the need to review the statins list. Multiattribute scoring tool (MAST) which takes into consideration all variables for rational decision making could help decision makers in reviewing the list. In this study, the six available statins were evaluated and scored.

## Materials and methods

Published literatures were studied and five sessions of expert group discussions were conducted to build the MAST. The attributes (and factors) identified for analysis were efficacy (clinical efficacy, clinical end points), safety (drug interactions, hazardous side-effects, documentation), drug applicability (drug strength/formulation, indications, dose frequency, side-effects, food interactions, dose adjustments) and costs. The average weights assigned by group members for efficacy, safety, drug applicability and costs were 32.6%, 26.2%, 24.1% and 17.1% respectively. Utility values of attributes were scored based on published evidences or/and agreements during group discussions. Attribute scores were summed to provide total utility score for each statin.

## Results

Atorvastatin scored the highest total utility score (TUS) of 77.88, followed by simvastatin (75.05). Atorvastatin and simvastatin scored consistently high even before acquisition costs were included. Low score on side effects for atorvastatin were compensated by higher scores on clinical end points resulting in higher TUS for atorvastatin. Pravastatin, lovastatin and rosuvastatin received TUS of 71.95, 71.45 and 63.74 respectively. Fluvastatin had the lowest score of 59.83.

## Conclusion

The multiattribute utility scoring tool successfully systematizes decision variables to aid selection of statins for the formulary. Based on total utility scores calculated using the designed MAST, atorvastatin and simvastatin should be considered as first-lines in the treatment of hypercholesterolaemia.

